# Hematoma expansion in intracerebral hemorrhage: neuroimaging biomarkers, radiomics-based prediction models, and clinical translation

**DOI:** 10.3389/fnhum.2026.1955696

**Published:** 2026-09-22

**Authors:** Haoliang Zhu, Tianyun Zhang, Hangzhe Sun, Yu Chen, Xingjian Sun, Bowen Wang, Yang Zhu, Kankai Wang, Anke Zhang, Yuanbo Pan

**Affiliations:** 1 The Second Affiliated Hospital, School of Medicine, Zhejiang University, Hangzhou, China; 2 The First Clinical Medical College, Wenzhou Medical University, Wenzhou, China; 3 National Clinical Research Center for Ocular Diseases, Eye Hospital, Wenzhou Medical University, Wenzhou, China; 4 Department of Neurosurgery, The First Affiliated Hospital of Bengbu Medical University, Bengbu, China; 5 Department of Neurosurgery, National Regional Medical Center, Binhai Campus of the First Affiliated Hospital, Fujian Medical University, Fuzhou, Fujian, China; 6 Department of Neurosurgery, The Second Affiliated Hospital, School of Medicine, Zhejiang University, Hangzhou, China; 7 Key Laboratory of Precise Treatment and Clinical Translational Research of Neurological Diseases, Hangzhou, China

**Keywords:** artificial intelligence, clinical translation, hematoma expansion, intracerebral hemorrhage, predictive models, radiomics

## Abstract

Intracerebral hemorrhage (ICH) is associated with substantial morbidity and mortality, and early hematoma expansion (HE), defined as an increase in hematoma volume on follow-up imaging, is a major determinant of early neurological deterioration and poor outcomes. Accurate, hyperacute prediction of HE is critical for guiding precise interventions, such as intensive blood pressure management and surgical evacuation. This narrative review examines the progression of prediction models from univariate predictors to systems incorporating artificial intelligence (AI), with particular attention to risk factors, model validation, and clinical translation. We further examine the validation status of current prediction models and the methodological and practical barriers to their clinical implementation. We review the progression from established independent predictors, such as the CT angiography (CTA) spot sign (SpS) and non-contrast CT (NCCT) island sign (IS), to multivariable clinical scoring systems, radiomics-based machine learning (ML) approaches, and deep learning (DL) models, while also discussing emerging generative architectures. Furthermore, major clinical trials evaluating strategies to limit HE are reviewed. Finally, the current challenges and future prospects of predictive models of HE are also elucidated.

## 1 Introduction

Stroke is a major global cause of death and disability. ICH accounts for approximately 10–20% of all stroke cases worldwide and is associated with high morbidity and mortality (Krishnamurthi et al., 2013; Morotti et al., 2023; Dowlatshahi et al., 2011a). ICH predominantly arises from the rupture of small arteries, leading to brain damage that is categorized into two distinct phases: primary and secondary injury. Primary brain damage occurs immediately at the time of vessel rupture, whereas secondary brain damage develops in a delayed manner following the primary damage (Keep et al., 2012; Shao et al., 2019; Mendelow and Crawford, 1997). The most common underlying cause of such vessel rupture is sporadic cerebral small vessel disease, which manifests in two principal forms: deep perforating artery vasculopathy and cerebral amyloid angiopathy (Charidimou et al., 2022; Rodrigues et al., 2018; Duering et al., 2023; Cordonnier et al., 2018). Clinically, ICH is characterized by an acute onset, manifesting as a spectrum of neurological deficits including hemiparesis, impaired consciousness, speech disturbances, and facial paralysis (Sheth, 2022). A primary driver of this poor prognosis is HE, which represents a critical and potentially modifiable therapeutic target.

HE is defined as a significant increase in hematoma volume on follow-up imaging relative to baseline. Epidemiological data suggest that approximately one-fifth to one-third of ICH patients experience HE within the first 24 h after onset (Chu et al., 2023; Li et al., 2020). HE is strongly associated with early neurological deterioration, increased intracranial pressure, mortality, and poor functional outcomes (Li et al., 2020; You et al., 2019). Crucially, the window for active intervention is narrow. HE predominantly occurs in the hyperacute phase, typically within 3 h of symptom onset (Wang et al., 2022). Therefore, rapid and accurate identification of early HE (EHE) upon hospital arrival is critical for effective hemostatic or surgical interventions.

Traditionally, EHE prediction has relied on the identification of independent risk factors through regression analyses. Clinical parameters related to EHE include the time from symptom onset to the initial brain imaging (<3 h) (Kazui et al., 1996; Brott et al., 1997; Dowlatshahi et al., 2016), blood glucose levels (Scott et al., 1999), systolic blood pressure (SBP ≥ 160 mm Hg) (Rodriguez-Luna et al., 2018), body temperature (Leira et al., 2004), baseline weight (Broderick et al., 2007), alcohol consumption (Fujii et al., 1998), age (age ≥ 85 years) (Forti et al., 2016), baseline CT time (within 48 h of onset), and follow-up CT time (within 120 h of onset) (Kazui et al., 1996; Brott et al., 1997; Dowlatshahi et al., 2016; Yao et al., 2015). In terms of neuroimaging, larger baseline hematoma volume and the “SpS” on CTA are well-established predictors (Kazui et al., 1996; Brott et al., 1997; Dowlatshahi et al., 2016), while NCCT markers [e.g., blend sign (BS) and IS] have more recently emerged as accessible alternatives to CTA (Huang et al., 2023; Li et al., 2017). However, these factors are typically analyzed as individual univariate predictors, which inherently limits their overall predictive specificity (SPE) and sensitivity (SEN) (An et al., 2017).

However, a critical disconnect remains: while interventions such as intensive blood pressure lowering and hemostatic therapies have successfully reduced EHE rates (e.g., in the TICH-2 trial), they have failed to improve functional outcomes (Sprigg et al., 2018). Since individual risk factors lack the SEN and SPE required for precise individual-level stratification, the field has shifted from univariate predictors to integrated prediction models. These range from simple clinical prediction scores (combining clinical and imaging variables) to sophisticated machine learning (ML) and radiomics approaches that mine high-dimensional quantitative features and finally to end-to-end deep learning (DL) algorithms capable of automated image analysis (Cheng et al., 2021; Zhu et al., 2021).

In this narrative review, we provide an overview of the landscape of EHE prediction. First, we clarify the heterogeneous definitions and standards of EHE used across ongoing and completed clinical trials. Then, we demonstrate independent predictors of EHE identified in regression studies and validated clinical prediction models. We summarize the reported predictive performance and validation characteristics of traditional clinical scores, radiomics-based models, ML approaches, and DL models while considering the substantial methodological heterogeneity across studies. Furthermore, we summarize key clinical trials that have utilized these EHE predictive markers for patient enrichment. Finally, we discuss the clinical translational gap and future directions for integrating these models into routine workflows to optimize ICH care.

A focused literature search was performed in PubMed for studies published through 31 January 2026, using combinations of terms related to ICH, HE, neuroimaging, radiomics, machine learning, deep learning, prediction models, validation, and clinical trials. Priority was given to original studies of HE predictors and prediction models, model development or validation studies, systematic reviews and meta-analyses, major clinical trials, and relevant methodological guidance. Studies primarily addressing traumatic hematoma progression, non-spontaneous ICH, or outcomes unrelated to HE prediction were generally excluded from the prediction-model synthesis.

## 2 HE

The primary challenge in accurately predicting HE lies in its heterogeneous definition (Dowlatshahi et al., 2011a). Although HE has reached a consensus on the concept (the increase in the volume of the hematoma on the images), there is still no standard for the specific definition. Fujii et al. initially defined HE as an absolute volume increase of >20 mL or a relative volume increase of >50% within 6 h of onset (Fujii et al., 1994). After that, Brott et al. suggested that a relative increase of more than 33% should be used as the standard for CT testing of HE (Brott et al., 1997). Kazui et al. suggested an alternative definition requiring a > 12.5 mL absolute increase or a > 40% relative increase (Kazui et al., 1996). The most common definition of HE in the literature right now uses a combination threshold, which is an increase in relative volume of more than 33% or an increase in absolute volume of more than 6 mL (some studies use >12.5 mL) (He et al., 2018). In addition, the differences in the methods used to measure the volume of the hematoma (e.g., the traditional ABC/2 formula, the Coniglobus formula, and the fully automated 3D reconstruction technique) made the results even more variable (Gunda et al., 2021; Huang et al., 2018; Huang and Yang, 2018).

These definitional discrepancies directly impact the fluctuations in HE report incidence and the comparability of different predictive model performances. To make this challenge more clear, Table 1 shows a summary of the HE definitions used in important clinical trials. Although there are differences, most recent clinical trials tend to use a relative volume increase of >33% as a core measure because it has been shown to be a reliable predictor of poor prognosis (He et al., 2018).

**Table 1:** Different definitions of HE in clinical trials.

Trial name	Intervention	Definition of HE	Timing of follow-up imaging/HE assessment	HE as outcome	Trial registration
INTERACT-1	Intensive blood pressure lowering	Relative growth ≥33% or absolute growth ≥12.5 mL	24 h	Primary	NCT00226096
INTERACT-2	Intensive blood pressure lowering	Relative growth ≥ 33% or absolute growth ≥12.5 ml	24 h	Secondary	NCT00716079
ATACH-2	Intensive blood pressure lowering	Relative growth ≥33%	24 h	Secondary	NCT01176565
TICH-2	Tranexamic acid (TXA)	Relative growth ≥33% or absolute growth ≥6 ml	24 h	Secondary	ISRCTN93732214
STOP-AUST	TXA	Relative growth ≥33% or absolute growth ≥6 ml	24 h	Primary	NCT01702636

In conclusion, this review primarily focuses on EHE, which is generally defined as hematoma growth occurring within 24 h of symptom onset or baseline CT. This period represents the most dynamic phase of hematoma evolution and is therefore of particular clinical relevance for acute interventions, including blood pressure control and anticoagulation reversal. In contrast, delayed HE represents a distinct phenomenon characterized by an increase in hematoma volume after the initial 24- to 48-h stabilization period. For example, in the SHRINC trial, delayed HE was defined as an increase in hematoma volume of >0.5 mL on MRI performed after 48 h (Gonzales et al., 2013). The mechanisms underlying delayed HE may differ from those of EHE and may involve rebleeding, coagulopathy, or inflammatory processes (Brouwers and Greenberg, 2013); however, delayed HE is beyond the scope of the present review, which focuses on prediction and intervention during the hyperacute and early acute phases.

## 3 Risk factors of HE

Risk factors of HE are primarily derived from clinical, imaging, and laboratory data (Figure 1; Table 2).

**Figure 1:**
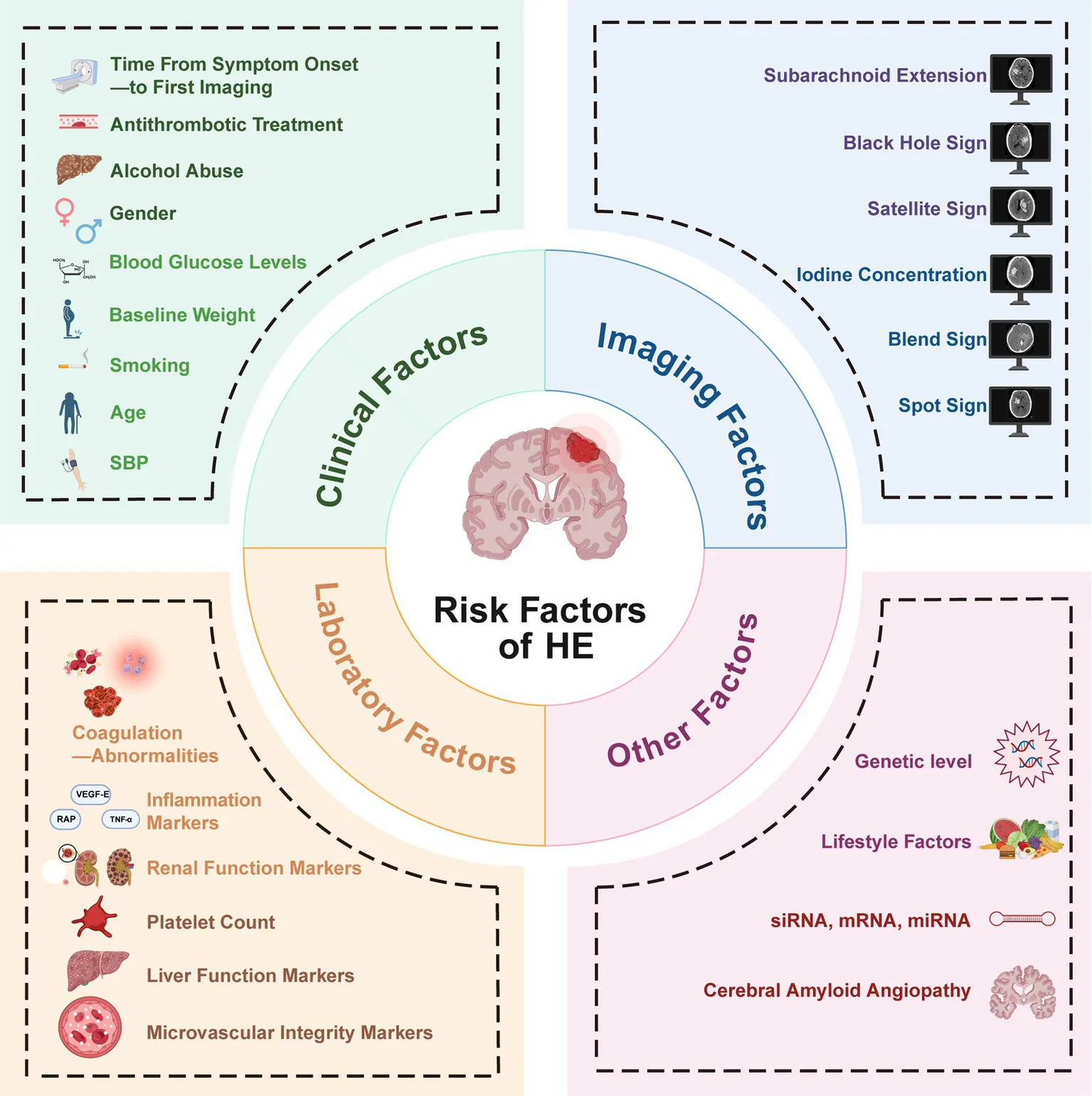
Risk factors associated with HE. Schematic overview of the major clinical, imaging, and laboratory factors associated with HE in patients with ICH. Clinical predictors include variables such as blood pressure, anticoagulant use, and onset-to-scan time. Imaging biomarkers derived from NCCT and CTA, including signs such as the BS, IS, and SpS, reflect hematoma instability and active bleeding. These factors collectively contribute to the risk stratification of HE.

**Table 2:** Independent predictors of HE validated by multivariable regression or meta-analysis.

Category	Predictor	Association with HE	Ref.
Time factors	Onset-to-scan time	Negative correlation (shorter time correlates with higher risk, especially <3 h or <6 h)	Kazui et al. (1996)
Clinical factors	Baseline hematoma volume	Positive correlation (larger volume correlates with higher risk)	Dowlatshahi et al. (2011a)
	Admission systolic blood pressure (SBP)	Positive correlation (higher blood pressure correlates with higher risk)	Rodriguez-Luna et al. (2018)
	Age	Positive correlation (advanced age is a risk factor)	Forti et al. (2016)
	Anticoagulation	Vitamin K antagonist (VKA, e.g., warfarin) use (strong correlation)	Flibotte et al. (2004)
		Direct oral anticoagulant (DOAC) use (correlated, lower risk than VKA)	Dowlatshahi et al. (2011a)
	Antiplatelet therapy	Inconsistent evidence (weak correlation in some meta-analyses)	Thompson et al. (2010)
	Blood glucose	Positive correlation (hyperglycemia is a risk factor)	Scott et al. (1999)
	Liver disease	Positive correlation	Parikh et al. (2020)
NCCT imaging	BS	Strong positive correlation	Sporns et al. (2017)
	IS	Strong positive correlation	Wei et al. (2020)
	Black hole sign (BHS)	Strong positive correlation	Chen et al. (2020)
	Subarachnoid hemorrhage extension (SAHE)	Strong positive correlation	Samarasekera et al. (2017)
CTA imaging	SpS	Consistently associated with increased HE risk; one of the most extensively validated imaging biomarkers	Li et al. (2011)
MRI imaging	“Spots” on SWI	Positive correlation (≥2 hypointense foci)	Allam et al. (2019)
	Cerebral microbleeds (CMBs)	Positive correlation (high CMB load)	Kidwell and Wintermark (2008)

### 3.1 Clinical factors

Multiple studies have validated numerous clinical predictors with robust predictive value for hematoma expansion (HE). In terms of medical history, a history of cerebral infarction (Kazui et al., 1997), alcohol consumption (Fujii et al., 1998), gender (Marini et al., 2017), age (Forti et al., 2016), and smoking history (Ariesen et al., 2003) are crucial factors. For baseline assessment, blood glucose levels (Scott et al., 1999), blood pressure (BP) (e.g., SBP (Rodriguez-Luna et al., 2018), blood pressure variability (Rodriguez-Luna et al., 2013), and mean arterial pressure), body temperature (Leira et al., 2004), and baseline weight (Broderick et al., 2007) are critical to HE. Importantly, a shorter interval from symptom onset to baseline CT, particularly imaging performed during the first several hours, is associated with a greater likelihood of subsequent HE (Kazui et al., 1996; Brott et al., 1997). Among these, SBP and anticoagulant use have been widely applied in clinical practice.

#### 3.1.1 Hypertension

Hypertension is the most important risk factor for spontaneous intracerebral hemorrhage (sICH). In acute ICH, 75% of patients present with SBP > 140 mmHg, and 20% have SBP exceeding 180 mmHg (Qureshi et al., 2007). Randomized trials have established the feasibility and general safety of early intensive blood pressure lowering in acute ICH, although its effects on hematoma growth and functional outcome have varied across studies. In the Intensive Blood Pressure Reduction in Acute Cerebral Hemorrhage II (INTERACT II), intensive SBP lowering did not significantly reduce the primary dichotomized outcome of death or major disability, although the prespecified ordinal analysis of the modified Rankin Scale favored intensive treatment (Yang et al., 2015; Qureshi, 2007). Recently, Li et al. proposed a lower target (130–139 mmHg), but this requires validation in large prospective trials before clinical implementation (Li et al., 2020). In addition, hypertension leads to a higher risk of deep ICH than of lobar ICH (adjusted relative risk: 25.9 versus 9.2) (Zia et al., 2007).

#### 3.1.2 Antiplatelet treatment and anticoagulation

Numerous studies have confirmed that antithrombotic therapy—encompassing antiplatelet treatment and anticoagulation—increases the risk of ICH. Clinically, the incidence of anticoagulation-related ICH is rising among elderly patients, driven by the growing use of oral anticoagulants (Flaherty et al., 2007). Thompson et al. showed that prior antiplatelet use was associated with higher post-ICH mortality and an elevated risk of EHE (Thompson et al., 2010).

#### 3.1.3 Onset-to-scan time

The time elapsed from symptom onset to the initial CT scan (onset-to-scan time) is a critical and independent predictor of HE. Contrary to some earlier misconceptions, a shorter onset-to-scan time (e.g., <3 or <6 h) is strongly associated with a higher risk of subsequent hematoma growth (Kazui et al., 1996; Brott et al., 1997; Dowlatshahi et al., 2016). A short interval (e.g., <3 h) implies that the initial CT scan has “captured” the hematoma during its most active, unstable phase, where active bleeding may persist due to unformed hemostatic plugs or high hydrostatic pressure. Therefore, patients scanned early are statistically more likely to exhibit expansion on follow-up imaging compared to those scanned later (e.g., >6 h), whose hematomas have likely already stabilized (Kazui et al., 1996). Even after adjusting for confounding variables such as baseline volume and the presence of the CTA SpS, early arrival remains an independent risk factor. Thus, onset-to-scan time serves not merely as a chronological metric but as a powerful surrogate marker for the dynamic instability of the hematoma, underscoring the urgency of hyperacute intervention.

#### 3.1.4 Other factors

Beyond the primary factors listed above, several other clinical variables significantly influence HE. Baseline hematoma volume is consistently reported as one of the strongest predictors; larger initial hematomas exert greater mechanical stress on surrounding tissues, creating a self-perpetuating cycle of expansion (Brouwers et al., 2014). Hyperglycemia on admission is another adverse prognostic factor, potentially exacerbating the inflammatory response and blood–brain barrier disruption (Yang et al., 2015). Furthermore, coagulopathy secondary to liver disease or renal dysfunction impairs the body’s intrinsic hemostatic capacity, thereby extending the duration of active bleeding and increasing the likelihood of significant expansion (Molshatzki et al., 2011).

### 3.2 Pathophysiology-informed imaging biomarkers of HE

The accurate prediction of HE relies on identifying specific neuroimaging features that reflect the underlying pathophysiological instability of the hematoma. To date, the widely accepted explanatory framework for this phenomenon is the “avalanche model”, proposed by Charles Miller Fisher. This model posits that the mass effect of the initial hemorrhage generates mechanical stress, leading to the shearing and rupture of adjacent pathological small vessels. This secondary bleeding contributes to the progressive growth of the hematoma, ultimately determining its final size (Gunda et al., 2021; Fisher, 1971).

Consequently, imaging biomarkers should be interpreted as macroscopic visualizations of these microscopic “avalanche” events. Advances in neuroimaging have facilitated the translation of these pathological concepts into clinical predictors. Imaging intervals used to quantify HE vary considerably across studies. In contemporary acute ICH studies, baseline imaging is generally obtained during the hyperacute phase and is commonly followed by repeat CT at approximately 24 h, although both baseline and follow-up timing differ across cohorts (Dowlatshahi et al., 2016; Yao et al., 2015).

Early investigations laid the groundwork for this field. For instance, Barras et al. demonstrated that fundamental hematoma morphological characteristics, particularly irregular shape and heterogeneous density, were independently associated with subsequent HE (Barras et al., 2009). These findings align with the avalanche model as irregularity often reflects uneven expansion into surrounding tissues. Subsequent research has further refined these observations into specific, standardized radiological signs on both NCCT and CTA. These include the well-validated SpS on CTA, which reflects active bleeding, as well as NCCT markers of hematoma heterogeneity (e.g., BHS and BS), all of which demonstrate significant predictive value (Huang et al., 2023). Figure 2 provides a timeline summarizing the chronological evolution of these key imaging predictors since their initial discovery.

**Figure 2:**
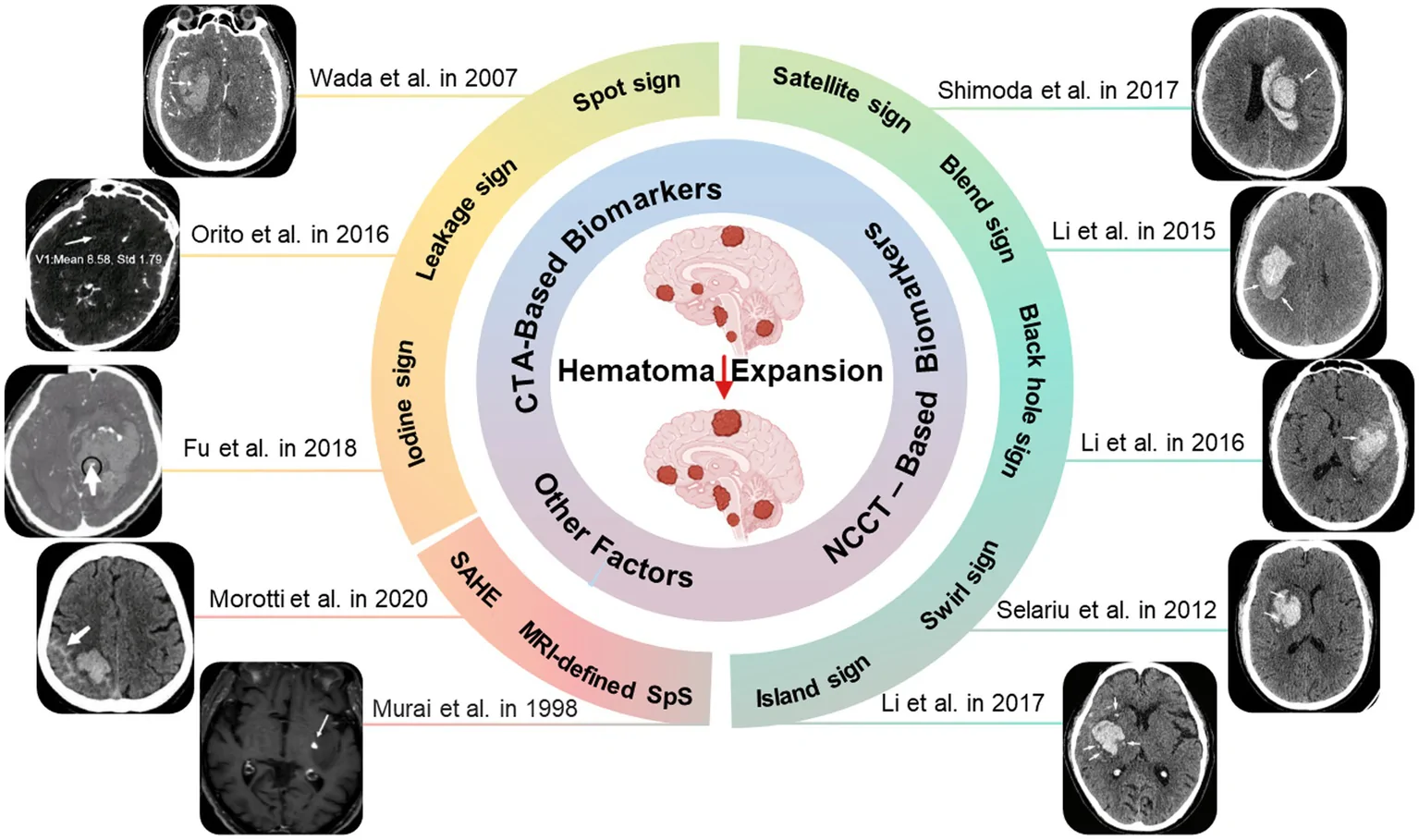
Imaging biomarkers associated with HE in ICH. Representative neuroimaging markers that have been reported to predict HE in ICH. CTA-based biomarkers include the SpS, leakage sign, and IoS, which reflect active contrast extravasation. NCCT-based markers such as the BS, BHS, IS, SaS, and swirl sign indicate hematoma heterogeneity and structural instability. These imaging features provide important visual indicators for identifying patients at high risk of early hematoma growth. Representative imaging panels were adapted from Ai et al. (2024), PeerJ 12:e17556, and Huang et al. (2023), Front. Neurol. 14:1176390, under the respective Creative Commons Attribution licenses, with modifications.

#### 3.2.1 CTA-based biomarkers

CTA combines CT imaging with intravenous contrast to rapidly visualize cerebral vasculature. Physiologically, CTA markers represent the most direct evidence of active contrast extravasation resulting from the rupture of small vessels or microaneurysms within the hematoma (d'Esterre et al., 2011).

##### 3.2.1.1 SpS

First described by Wada et al. (2007), the SpS was defined as one or more enhancing foci (1–2 mm in diameter). The SpS represents the precise site of active bleeding or “secondary vessel rupture” described in the avalanche model. It reflects ongoing arterial bleeding into the hematoma cavity, indicating that the hemostatic plug has not yet formed or has failed due to coagulation abnormalities. Currently, SpS is recognized as one of the most effective imaging markers for hematoma expansion (HE) prediction. Demchuk et al. reported that the SpS has a SEN of 51.0%, SPE of 85.0%, positive predictive value of 61.0%, and negative predictive value of 78.0% for HE (Demchuk et al., 2012). Furthermore, Delgado et al. proposed a score system for quantifying SpS, including the number of SpS, maximum attenuation, and maximum size (Delgado Almandoz et al., 2009). This system not only predicts EHE but also achieves accurate classifications on HE, in-hospital mortality, and clinical outcomes (Li et al., 2011). In addition, SpS is associated with the presence of mortality and neurological deterioration (Wada et al., 2007; Xu et al., 2018), early clinical manifestations, coagulation status, use of anticoagulants, Glasgow Coma Scale (GCS) score at onset, mean arterial pressure >120 mmHg, and intraventricular hemorrhage (IVH) (Delgado Almandoz et al., 2009; Romero et al., 2013).

##### 3.2.1.2 Leakage sign

Although the SpS is relatively specific, its SEN is limited. To identify more subtle contrast leakage that may reflect ongoing bleeding, Orito et al. proposed the leakage sign (LS) (Orito et al., 2016). Researchers placed a 1-cm-diameter region of interest (ROI) over the area of maximal Hounsfield unit (HU) change on the delayed CTA phase image. They then transferred the identical ROI to the corresponding location on the arterial-phase CTA image. A positive LS was diagnosed if the delayed-phase ROI showed a 10% HU increase compared to the arterial-phase measurement. A positive LS was associated with significantly worse prognosis (*p* = 0.03). The results showed that LS had higher SEN (93.30%) and SPE (88.80%) than SpS for HE prediction.

##### 3.2.1.3 Iodine sign

IoS, proposed by Fu et al., serves as a dynamic marker for contrast leakage and HE prediction (Fu et al., 2018). As a novel marker enabled by gemstone spectral imaging, it allows direct quantitative assessment of iodine concentration (IC) via monochromatic image reconstruction. A positive IoS is defined by (1) visual identification of ≥1 intralesional enhancement, (2) IC > 782 μg/mL within the ROI, and (3) anatomical discontinuity from surrounding vasculature. A subsequent study reported a SEN of 78.7% and an AUC of 0.824 for EHE prediction (Shakya et al., 2021).

#### 3.2.2 NCCT-based biomarkers

Validated NCCT markers, including shape- and density-related signs, most notably the IS, SaS, BS, and BHS, serve as surrogates for hematoma heterogeneity. Pathophysiologically, these signs reflect the mechanical consequences of the initial hemorrhage’s mass effect on surrounding tissues, consistent with the secondary rupture of peripheral vessels (Blacquiere et al., 2015; Broderick et al., 1993; Dowlatshahi et al., 2011b; Boulouis et al., 2017).

##### 3.2.2.1 Shape-related signs

###### 3.2.2.1.1 IS

Proposed by Li et al. (2017), the IS is defined as the presence of ≥ 3 discrete hematomas surrounding the main bleeding site or ≥ 4 scattered small hematomas. IS serves as an independent predictor of EHE and is widely incorporated in HE risk scoring systems. Pathophysiologically, these “islands” likely represent distinct foci of secondary bleeding from separate arterioles that have ruptured due to the pressure wave of the main hematoma. While its incidence is relatively low (approximately 21%), a meta-analysis by Wei et al. demonstrated that the IS exhibits high SPE (89%) with an AUC of 0.73, making it an independent and reliable predictor of early expansion (Wei et al., 2020).

###### 3.2.2.1.2 Satellite sign

Similar to the IS, SaS describes a small hemorrhagic focus located in the perihematomal region, detached from the main hematoma body. First identified by Shimoda et al., the SaS is observed more frequently than the IS (incidence of 49.2%) but correlates with a slightly lower rate of absolute expansion (Shimoda et al., 2017). A meta-analysis showed that the SaS could effectively predict HE with a SEN of 50% and a SPE of 71% (Yang et al., 2020). Like the IS, the SaS suggests that mechanical stress has compromised vessels in the immediate periphery, creating a satellite bleed that carries a risk of merging with the main hematoma.

##### 3.2.2.2 Density-related signs

###### 3.2.2.2.1 BS

BS, first characterized by Li et al. (2015), refers to a hypoattenuating area adjacent to a hyperattenuating region with a sharp margin and density difference ≥ 18 HU. It reflects heterogeneous blood stages and rebleeding and further contributes to HE. In a study of 172 ICH patients, the incidence of BS was 16.9%, with a high SPE (95.5%) for predicting HE (Sporns et al., 2017). This imaging marker is valuable for identifying patients at high risk of HE.

###### 3.2.2.2.2 BHS

BHS, first reported by Li et al., is characterized by a well-defined hypodense region surrounded by hyperdense hematoma, with no connection to adjacent brain parenchyma (Li et al., 2016). It is defined by CT attenuation difference ≥ 28 HU between the hypo- and hyperdense regions. In a cohort study, the incidence of BHS was 14.6%, with a SEN of 31.9% and SPE of 94.1% for predicting EHE. Pathophysiologically, the “black hole” suggests a core of fresh, unclotted blood indicating a very recent bleed within the hematoma structure. A meta-analysis confirmed its strong SPE (93%) and AUC (0.83), and it reinforces its role in reflecting hematoma heterogeneity and HE risk (Chen et al., 2020). By analyzing hematoma heterogeneity, the BHS reflects intralesional bleeding at different stages to predict subsequent HE risk.

###### 3.2.2.2.3 Swirl sign

SwS is characterized by one or more regions of hypoattenuation or isoattenuation within a hyperattenuated hematoma, including round, streak-like, or irregular shapes (Ng et al., 2018). First formally described by Selariu et al., the SwS has been investigated for its prognostic value in predicting HE (Selariu et al., 2012). Meta-analyses have reported moderate SEN (45–50%) and relatively high SPE (77–79%) for predicting HE (Yu et al., 2019). While it shares similarities with other density markers, the swirl sign visualizes the unstable nature of the clot and the active influx of blood, providing a specific morphological target for risk stratification.

##### 3.2.2.3 Others

###### 3.2.2.3.1 SAHE

SAHE, defined as the presence of subarachnoid blood adjacent to an intraparenchymal hematoma, has been proposed as a HE predictor. SAHE may show the existence of weak vessels as well as active bleeding in the hematoma surrounding the vessels, lending credence to the “avalanche” hypothesis of HE (Suzuki et al., 2019). A multivariate study demonstrated that SAHE independently predicted the probability of HE in patients with lobar ICH after controlling for confounding variables (Samarasekera et al., 2017). Beyond these HE-specific signs, IVH topography has also been explored as a prognostic imaging biomarker in ICH (Chen et al., 2026b), and IVH itself encompasses heterogeneous structural etiologies (Chen et al., 2025).

#### 3.2.3 MRI-defined SpS

Beyond CT, recent studies have further introduced MRI-defined spot SpS, expanding the imaging spectrum of HE predictors. The SpS on MRI was first proposed by Murai et al. (1998). Early reports described hyperintense signals within the hematoma on post-contrast T1-weighted images, which were interpreted as evidence of contrast-medium extravasation. Subsequently defined by Valyraki et al., the MRI SpS appears as a spot-like or serpiginous high signal intensity (>1.5 mm) within the hematoma margin on contrast-enhanced scans, without connection to external vessels (Valyraki et al., 2023). A study reported that ≥2 spots on susceptibility-weighted imaging are associated with poor 3-month outcomes (Allam et al., 2019). Although less commonly used than CT markers due to acquisition time, these MRI biomarkers offer critical insights into the microvascular environment and warrant further research to establish their routine clinical utility.

### 3.3 Comparative weighting of predictors and clinical implications

Although several clinical and imaging variables are consistently associated with HE, their reported effect sizes should not be interpreted as establishing a universal hierarchy of predictor importance. Apparent associations vary according to patient selection, HE definition, imaging timing, covariate adjustment, and the combination of predictors included in each multivariable model. The CTA SpS remains one of the most extensively validated imaging biomarkers of active bleeding, whereas shorter onset-to-scan time, baseline hematoma volume, anticoagulant exposure, and NCCT markers also contribute predictive information in different settings. Accordingly, effect estimates derived from separate studies should be interpreted within their respective methodological contexts rather than ranked directly across studies.

The clinical implication of these weight disparities is profound. While traditional scoring systems offer interpretability, they often rely heavily on dichotomous markers, potentially oversimplifying risk by neglecting the predictive granularity of continuous variables. To address this, future model development should prioritize “weight transparency” through the use of standardized regression coefficients, feature importance ranking, or SHapley Additive exPlanations (SHAP) values (Tanioka et al., 2022). Furthermore, establishing stratified clinical decision pathways based on these weight hierarchies is essential. In clinical practice, high-risk predictions may be more appropriately used to prioritize closer neurological monitoring, repeat imaging when clinically indicated, expedited multidisciplinary assessment, or enrollment into biomarker-enriched trials. Whether model-defined risk thresholds should trigger a specific hemostatic intervention requires prospective evaluation in treatment-impact studies. Finally, given the complexity of these interactions, there is an urgent need for multicenter studies to validate the external reproducibility of ML-derived weights, ensuring that predictive models are robust against overfitting and spurious associations (Gregório et al., 2018).

## 4 Prediction models of HE

Prediction models (also referred to as prognostic models or scoring systems) integrate multiple risk factors (clinical and imaging factors) to estimate the risk of HE (Collins et al., 2015). While simple clinical scores such as the GCS and National Institutes of Health Stroke Scale (NIHSS) are widely used for general stroke assessment, specific HE prediction models have struggled with widespread adoption due to either suboptimal performance or cumbersome application (Thompson et al., 2014). To provide a clear landscape of the current tools, we categorize existing models into three methodologically distinct groups: traditional clinical prediction scores, ML-based models, and DL models. We summarize the architectural designs, validation cohorts, and performance metrics of these models in Tables 3, 4. The applicability of conventional ICH functional scores to mild-to-moderate hemorrhage has also been specifically examined (Chen et al., 2026a).

**Table 3:** Traditional clinical prediction models and scores.

Author [Ref.]	Time	Model name	Research design	Predictive factors (inputs)	Train (*n*)	Val. (*n*)	Train AUC	Val. AUC
Takeda et al. (2013)	2007–2009	HE prediction model	Single center, retrospective	NCCT + Clinical (Initial SBP, 1.5 h-SBP)	201	N/A	0.913	N/A
Miyahara et al. (2018)	2018	HEAVN score	Single center, retrospective	NCCT (Hypodensity) + Clinical (Age, Vol, SBP, Time)	457	165	0.81	0.8
Bakar et al. (2021)	2015–2020	HEMRICH scale	Multicenter, retrospective	NCCT + Clinical (GCS, Vol)	46	N/A	0.83	N/A
Hu et al. (2021)	2012–2018	Nomogram	Single center, retrospective	NCCT (Density, Vol) + Clinical	477	N/A	0.883	N/A
Yu et al. (2021)	2017–2021	Nomogram (HTN-ICH)	Single center, retrospective	NCCT (SwS, BS, Irreg) + Clinical (Vol, Time)	341	N/A	0.762	N/A
Zhang et al. (2022)	2008–2021	Predictive nomogram	Single center, retrospective	NCCT (Blend, SwS) + Clinical (GCS)	506	103	0.908	0.844
Bo et al. (2023)	2015–2020	Hybrid model	Multicenter, retrospective	NCCT + Clinical (Vol, Prothrombin time)	204	204	0.949	N/A
Li et al. (2023)	2023	Prediction score table	Single center, retrospective	NCCT (Island, Blend) + Clinical (Vol, GCS)	44 (HE)	N/A	0.858	N/A
Rodriguez-Luna et al. (2023)	2013–2021	CTA/NCCT scores	Single center, prospective	NCCT/CTA + Clinical (Age, Vol, GCS)	132	105	0.69	0.59
Chen Y. et al. (2024)	2014–2024	SwS score System	Multicenter, retrospective	NCCT (Swirl Sign) + Clinical (Time)	160	71	0.773	0.77

Performance metrics are reported as published in the original studies. Because the included studies differ in patient case mix, HE definitions, imaging protocols, feature engineering, and validation strategies, AUC values should not be interpreted as directly comparable across rows.

**Table 4:** Predictive models based on ML and DL.

Author [Ref.]	Model name	Research design	Input features (predictors of HE)	Train (*n*)	Val. (*n*)	AUC (HE)
Part A: ML models						
Chen et al. (2021)	Clinical-radiomics	Single center, retrospective	Radiomics (Intensity) + Clinical	864	289	0.82 (Val)
Feng et al. (2024)	Combined model	Multicenter, retrospective	Radiomics (Wavelet) + Clinical	64(HE)	181	0.82 (Val)
Xia et al. (2022)	Clinical-semantic	Double center, retrospective	Radiomics + BRAIN score	299	77	0.94
Du et al. (2024)	XGBoost model	Multicenter, retrospective	NCCT (Island, Blend) + Gender	122(HE)	N/A	0.96
Zhou et al. (2021)	Modified BAT (ML)	Multicenter, retrospective	NCCT (Blend) + Clinical	162	70	0.83
Li Y. et al. (2024)	XGBoost	Single center, retrospective	CT extraction + Clinical	464	118	0.82
Dai et al. (2023)	Radiomics nomogram	Single center, cohort	NCCT (Island, Blend) + GCS	130	57	0.89
Zhang et al. (2022)	Radiomics model	Single center, retrospective	Radiomics (SwS) + Clinical	66	22	0.899
Part B: DL models						
Cheng et al. (2021)	Dual-Modal (ResNet)	Single center, retrospective	NCCT Images + Clinical	105	25	0.91
Ma et al. (2022)	U-Net + ResNet-34	Single center, cohort	NCCT Images + Clinical	57(HE)	N/A	0.927
Lu et al. (2024)	CNN-RNN	Single center, retrospective	2D/3D Images + Radiomics	180	100	0.95
Wu et al. (2024)	VGG-19	Single center, retrospective	Deep features (VGG) + Clinical	204	50	0.921
Tran et al. (2024)	Uncertainty-aware	Multicenter, retrospective	Radiomics (SwS) + Time	634	159	0.81
Lee et al. (2024)	Multitask DL	Single center, retrospective	NCCT Features + GCS	114(HE)	N/A	0.76
Yalcin et al. (2024)	EfficientNet	Single center, retrospective	CT + Synthetic Images	122	5-fold cross-validation	N/R

Performance metrics are reported as published in the original studies. Because the included studies differ in patient case mix, HE definitions, imaging protocols, feature engineering, and validation strategies, AUC values should not be interpreted as directly comparable across rows.

### 4.1 Traditional clinical prediction scores

The foundation of HE prediction lies in the identification of independent imaging predictors derived from NCCT and CTA, such as the BHS, IS, and BS. Traditional scoring systems aim to translate these qualitative signs and clinical variables into semi-quantitative risk scores (Table 3).

Early efforts focused on NCCT-based scoring systems. Morotti et al. developed the BAT score (BS, intrahematoma hypodensity, and time <2.5 h), which demonstrated high SPE (0.89) but moderate SEN (0.50), reflecting a conservative prediction capability (Morotti et al., 2018). To enhance SEN, subsequent models incorporated volumetric and outcome data. The HEMRICH scale integrated clinical and radiological variables and reported an AUC of 0.830 in its study cohort (Bakar et al., 2021). Similarly, Fu et al. proposed a 10-point scale combining volume thresholds (>30 mL), onset-to-CT time, and morphological irregularity, achieving a remarkable SEN of 97.8% (Fu et al., 2019).

With the incorporation of contrast-enhanced imaging, CTA-based models have added markers of active contrast extravasation and have demonstrated useful discrimination in individual cohorts. Rodriguez et al. confirmed that CTA features, particularly from multiphase protocols, offer superior discrimination (C-index: 0.79) than NCCT alone (C-index: 0.69) (Rodriguez-Luna et al., 2023). Building on this, Brouwers et al. constructed a 9-point score integrating the SpS with warfarin use and onset time (Brouwers et al., 2014), which was later expanded by Huynh et al. into the PREDICT A/B scores. While the PREDICT scores showed improved discrimination (AUC: 0.755), they highlight the persistent need for extensive external validation (Huynh et al., 2015).

More recently, nomograms have been employed to visualize logistic regression models, offering a user-friendly interface for clinicians. Yu et al. and Zhang et al. developed NCCT-based nomograms in hypertensive and severe ICH cohorts, respectively, reporting development-cohort AUCs of 0.762 and 0.908 (Yu et al., 2021; Zhang et al., 2022). Related nomogram approaches have also been developed for early acute hydrocephalus after spontaneous ICH (Chen A. et al., 2024).

While imaging-clinical hybrid scores improve performance by leveraging interpretable signs, they remain fundamentally limited by their reliance on visual interpretation. This introduces inter-observer variability and fails to capture sub-visual heterogeneity, necessitating the shift toward fully quantitative approaches.

### 4.2 Machine learning models

To overcome the subjectivity of manual scoring, radiomics has emerged as a paradigm shift, converting medical images into high-dimensional, mineable data (Mayerhoefer et al., 2020). Unlike traditional scores, ML models learn non-linear relationships from these quantitative features to enhance predictive precision (Greener et al., 2022). The standard radiomics workflow involves image acquisition, region of interest (ROI) segmentation, high-throughput feature extraction (capturing texture, shape, and intensity), and model construction using classifiers such as logistic regression (LR), support vector machine (SVM), and XGBoost (Rossi et al., 2021; Mu et al., 2020).

Initial studies demonstrated the power of radiomics signatures. Ma et al. developed a radiomics-based model for hypertensive ICH and reported an AUC of 0.892, suggesting that quantitative imaging features may provide useful predictive information beyond visually assessed CT characteristics (Ma et al., 2019). In a separate study, Xie et al. reported an AUC of 0.954 for a Least Absolute Shrinkage and Selection Operator (LASSO)-based radiomics model and observed better performance than the conventional radiological model evaluated within the same cohort (Xie et al., 2020).

However, the field is increasingly moving toward algorithm comparison and optimization. Duan et al. compared several classifiers within the same radiomics dataset and found that predictive performance varied across algorithms, with SVM and k-NN among the better-performing approaches in that study (Duan et al., 2022). Conversely, Tanioka et al. noted that without rigorous feature engineering, some ML models might only offer modest improvements over traditional scores such as the BAT score, cautioning against the blind application of algorithms (Tanioka et al., 2022).

Subsequent studies shifted toward the integration of clinical variables. Liu et al. employed SVM to combine clinical data (e.g., time from symptom onset to initial CT scan), laboratory indices (e.g., platelet and hemoglobin), and radiological features (Liu et al., 2019). Their model achieved a mean AUC of 0.89, highlighting the advantages of multimodal data integration. Furthermore, a clinical–radiomics nomogram was established (Chen et al., 2021), which incorporated radiomic features and clinical predictors (e.g., sex, GCS score, and time to baseline NCCT). The hybrid model achieved an AUC of 0.820 in the validation cohort and performed better than models using clinical or radiomics features alone. Du et al. incorporated NCCT features (e.g., IS and BS) and clinical variables (e.g., time to baseline CT, age, and gender) into models built with XGBoost, support vector machine (SVM), random forest (RF), and logistic regression (LR) (Du et al., 2024). In future clinical practice, scoring systems could be integrated into high-performance radiomics-clinical models. These models hold promise for delivering real-time, scalable, and individualized HE risk predictions to guide therapeutic decisions and trial enrollment.

### 4.3 Deep learning models

The frontier of HE prediction lies in DL and multimodal fusion, which aim to synergize the strengths of clinical priors, radiomic textures, and automated feature learning.

DL is a branch of ML that allows computing models with multiple processing layers to learn data representations at numerous abstraction levels (LeCun et al., 2015). The convolutional neural network (CNN) is the most commonly used DL architecture type in medical image analysis (Chartrand et al., 2017). Suggestions that CNN-extracted features can provide various high-order features of images and apply them to specific clinical outcomes exist (Liu et al., 2022). Successful application of DL requires a large number of training sets. However, medical datasets are often limited in number. Because medical imaging datasets are often limited in size, transfer learning is commonly used to initialize CNNs with weights pretrained on larger datasets, such as ImageNet, thereby reducing the amount of task-specific training data required (Bo et al., 2021).

Unlike radiomics, DL models (such as CNNs) perform end-to-end feature learning. Cheng et al. exemplified this by developing a dual-modal strategy: a deep CNN for image analysis combined with a multi-layer perceptron (MLP) for clinical data. This fusion significantly boosted accuracy to 86.5%, compared to 69.5% for the image-only model, confirming that clinical context (e.g., blood pressure and onset time) acts as a crucial regularizer for imaging models (Cheng et al., 2021).

Recent trends emphasize the construction of hybrid/joint models that integrate clinical demographics, radiological signs, and radiomics/DL features. This holistic approach addresses the “black box” nature of pure AI models by grounding them in clinical reality. Rezaei et al. demonstrated this using a random forest framework on basal ganglia hemorrhage, where a hybrid model incorporating 10 key radiomic features achieved an AUC of 0.90, vastly outperforming models relying solely on clinical (AUC: ~0.5–0.6) or simple imaging data (Rezaei et al., 2023). For instance, Xu et al. developed a nomogram for EHE risk assessment in 388 ICH patients (Xu et al., 2022). All patients received baseline NCCT within 6 h after onset and follow-up CT within 24 h. The team constructed five models: clinical, imaging, clinical-radiological, radiomics, and a joint model by using LASSO or stepwise LR. They validated these models in separate training (*n* = 235) and testing (*n* = 153) cohorts. The joint model, which combined clinical characteristics, radiological signs, and radiomics features, achieved a training AUC of 0.900 with 83.87% SEN and a testing AUC of 0.850 with 80.10% SEN. The resulting nomogram provides an interpretable, non-invasive NCCT-based tool for individualized risk stratification.

Reported performance metrics should be interpreted in the methodological context of each individual study rather than compared directly across studies. The studies summarized in Tables 3, 4 differ substantially in sample size and case mix, patient selection, definitions of HE and follow-up intervals, imaging acquisition and reconstruction protocols, segmentation and preprocessing procedures, feature selection strategies, and validation design. In particular, performance estimated in development cohorts, random internal splits, or cross-validation may be optimistic relative to that observed in geographically or temporally independent populations. Case mix can also materially influence discrimination, whereas outcome prevalence affects predictive values and other threshold-dependent metrics. Accordingly, higher AUCs reported in individual radiomics, ML, or DL studies should not be interpreted as evidence of superiority over conventional prediction scores unless the models are evaluated head-to-head in the same population using harmonized outcome definitions and validation procedures. Future studies should report uncertainty around discrimination estimates and evaluate calibration and external validity alongside AUC.

## 5 Clinical trials

Despite significant advances in clinical research, the precise pathophysiology of HE remains incompletely understood, creating a major bottleneck in translating therapeutic interventions into functional recovery (Xu et al., 2023; Xu et al., 2025). The optimal pharmacological strategy for preventing HE constitutes a central controversy in neurology. As detailed in Table 5, randomized controlled trials over the past decade have evolved from broad physiological management (e.g., intensive blood pressure reduction in INTERACT-2 and ATACH-2) to targeted hemostatic therapies (e.g., TXA in TICH-2 and rFVIIa). While many of these interventions, such as intensive BP lowering and rFVIIa, have successfully demonstrated a reduction in HE, a persistent “translational gap” remains: The restriction of HE rarely translates into significant improvement in long-term functional outcomes or survival. Consequently, recent trial designs (e.g., STOP-AUST and FASTEST) have shifted toward precision medicine, utilizing neuroimaging biomarkers (such as the CTA SpS) and ultra-early time windows to identify and enroll “active bleeders” who are most likely to benefit from aggressive intervention.

**Table 5:** Clinical trials targeting HE.

Trial (year, n)	Intervention	Marker/enrichment	Effect on HE and outcome	Trial registration
INTERACT-2 (2013, 2,839)	Intensive BP lowering (SBP < 140 mmHg)	Clinical: Hypertension; exclude massive hematoma	No clear reduction in HE; the primary dichotomized functional outcome was not significantly improved, although ordinal mRS analysis favored intensive BP lowering.	NCT00716079
ATACH-2 (2016, 1,000)	Intensive BP lowering (SBP 110–139 mmHg)	Clinical: Initial hematoma volume < 60 mL	Trend of reduced HE (18.9% vs. 24.4%, *p* = 0.08); no functional outcome improvement	NCT01176565
STOP-AUST (2019, 100)	TXA	Imaging: CTA SpS positive	No significant reduction in hematoma growth (adjusted OR 0.72, 95% CI 0.32–1.59; *p* = 0.41).	NCT01702636
TICH-2 (2018, 2,325)	TXA	confirmed ICH, symptom onset < 8 h	Reduced HE (25.1% vs. 28.7%; adjusted OR 0.80, 95% CI 0.66–0.98; *p* = 0.030); no improvement in 90-day functional outcome.	ISRCTN93732214
rFVIIa Phase III/FAST trial (2008, *n* = 841)	Recombinant factor VIIa	Clinical + Time window: Onset < 4 h; exclude coagulopathy	Reduced hematoma growth, particularly with 80 μg/kg rFVIIa, but did not improve survival or functional outcome; arterial thromboembolic events were increased in the 80 μg/kg group.	NCT00127283
FASTEST Part 1 (2026, 626)	Recombinant factor VIIa	Imaging: CTA SpS positive or NCCT features	Reduced 24-h hematoma growth but did not improve overall functional outcome; life-threatening thromboembolic events were more frequent with rFVIIa.	NCT03496883
TRANSACT (Ongoing, 326)	(TXA)	Symptom onset < 4.5 h	Ongoing; primary endpoint includes 24-h HE	NCT03044184

HE, hematoma expansion; SBP, systolic blood pressure; SpS, spot sign; TXA, tranexamic acid.

### 5.1 Intensive blood pressure reduction

The pilot INTERACT study (NCT00226096) provided early proof-of-concept evidence that intensive blood pressure lowering may attenuate hematoma growth. The subsequent INTERACT2 trial (NCT00716079) evaluated intensive SBP lowering in a substantially larger population. Although the primary dichotomized outcome of death or major disability did not reach statistical significance, a prespecified ordinal analysis of the modified Rankin Scale favored intensive treatment. These findings illustrate an important distinction between reducing hematoma growth or improving the overall distribution of functional outcomes and demonstrating benefit on a prespecified dichotomous clinical endpoint (Anderson et al., 2008).

### 5.2 Hemostatic therapies (TXA and factor VIIa)

Pharmacological limitation of HE has focused on hemostasis. The TICH-2 trial evaluated TXA, an antifibrinolytic agent, administered within 8 h of onset (Sprigg et al., 2018). Consistent with BP trials, TXA significantly reduced HE rates (*p* = 0.03) and early mortality (*p* = 0.04) but failed to improve 90-day functional status (adjusted OR: 0.88, *p* = 0.11). Earlier trials with recombinant activated factor VII (rFVIIa) demonstrated a robust reduction in hematoma growth but failed to improve survival, partly due to thromboembolic complications. These failures have shifted the field toward precision medicine, using predictive markers to identify the specific subgroup of “active bleeders” who would derive the greatest benefit from aggressive hemostatic interventions.

### 5.3 Imaging-guided and biomarker-enriched trials

Modern ICH clinical trials have focused on enrichment strategies. The STOP-AUST trial (CTA-guided) was a landmark phase II study that used the CTA SpS to screen patients and evaluate the efficacy of TXA (Meretoja et al., 2014). Although STOP-AUST did not demonstrate a statistically significant reduction in hematoma growth (adjusted OR: 0.72, 95% CI: 0.32–1.59; *p* = 0.41), its modest sample size and treatment timing may have limited the ability to detect a treatment effect. The FASTEST trial subsequently evaluated ultra-early rFVIIa treatment within 2 h of ICH onset. Part 1 results published in 2026 showed that rFVIIa reduced hematoma growth but did not improve overall functional outcome while increasing the risk of life-threatening thromboembolic events. Prespecified subgroup signals in patients treated particularly early and in those with a CTA SpS have informed the ongoing enriched FASTEST Part 2 strategy (Broderick et al., 2026). To overcome the critical time dependence of HE, trials such as TRANSACT are using mobile stroke units (MSUs) to administer TXA treatment in the ultra-early stage (<2 h, usually pre-hospital) (Cooley et al., 2021).

## 6 Prospects and challenges

HE is a critical driver of early neurological decline and unfavorable outcomes. Therefore, accurate risk stratification is essential to guide precise interventions. Radiomics and ML models have demonstrated promising discrimination in individual studies; however, an apparent performance advantage over conventional clinical or imaging models cannot be inferred from cross-study comparisons because of substantial clinical and methodological heterogeneity. Unlike the standardized workflows established for ischemic stroke, the integration of HE prediction models into routine ICH care faces significant translational hurdles.

ML models based on clinical and radiomics features hold the potential to optimize critical decision-making, such as BP management targets. However, algorithms alone cannot improve outcomes. As highlighted in the recent “Code ICH” consensus (Li Q. et al., 2024), optimal ICH care requires coordinated multidisciplinary action rather than isolated imaging evaluations. To enhance clinical relevance, we propose that predictive models should be integrated into a workflow. In such a framework, an automated HE-risk estimate could function as a triage aid, prompting expedited neurological reassessment, repeat imaging when clinically appropriate, and early multidisciplinary review. Model-derived risk estimates should support rather than replace clinical judgment, and thresholds for initiating specific anti-expansion therapies require prospective validation in treatment-impact studies. This integration requires defining clear decision thresholds, such as using high-specificity markers to rule in aggressive monitoring. Related studies have also evaluated whether preventive external ventricular drainage benefits patients with mild ventricular hemorrhage (Chen et al., 2023).

Despite the theoretical benefits, several barriers limit the widespread adoption of these models in real-time practice. (1) Time Constraints: HE is a highly dynamic process, most commonly occurring within the first 24 h, with the steepest volume increase in the first 6 h. Current radiomics workflows often involve complex steps (image transfer, ROI delineation, and feature extraction) that may delay decision-making. (2) Standardization Issues: As noted previously, the definition of HE varies across studies (e.g., inclusion of IVH) (Dowlatshahi et al., 2018), and imaging acquisition heterogeneity (e.g., slice thickness) affects feature reproducibility. (3) Workflow Disruption: Implementing complex DL models requires robust IT infrastructure. The “black box” nature of some DL algorithms also creates a trust barrier for clinicians who need interpretable rationale (e.g., visualizing the specific region of risk) before altering treatment plans.

Currently, no single AI model is ready for unassisted widespread clinical use. Beyond discrimination, clinical implementation requires evidence that predicted probabilities are accurate, transportable, interpretable, and clinically useful. Evaluation and reporting should also be aligned with contemporary prediction-model frameworks. The TRIPOD+AI statement provides updated guidance for transparent reporting of clinical prediction models developed using either regression or machine learning methods, whereas the updated PROBAST+AI framework provides structured criteria for assessing methodological quality, risk of bias, and applicability. In current HE prediction research, however, model evaluation remains dominated by discrimination metrics, particularly the AUC, while calibration, prespecified decision thresholds, net clinical benefit or decision-curve analysis, and prospective assessment of workflow impact remain inconsistently reported (Moons et al., 2025; Collins et al., 2024). These dimensions are essential for determining whether apparently accurate models can support clinically meaningful decisions. First, prediction models should undergo external validation in geographically and temporally independent cohorts and subsequently be evaluated prospectively within real-world clinical workflows to assess robustness across institutions, scanners, acquisition protocols, and patient case mix. Second, calibration should be evaluated alongside discrimination because a model may distinguish patients with and without subsequent HE while still systematically overestimating or underestimating an individual patient’s absolute risk. Calibration plots and appropriate quantitative calibration measures should therefore be reported whenever absolute risk estimates are intended to inform clinical decisions. Third, interpretability should extend beyond *post-hoc* feature-importance displays to include clinically coherent outputs, characterization of prediction uncertainty, and analysis of model failure modes. Finally, clinical utility should be assessed at prespecified decision thresholds using decision-analytic approaches such as net benefit or decision-curve analysis. Ultimately, prospective impact studies are needed to determine whether model-guided risk stratification improves treatment selection, workflow efficiency, or patient-centered outcomes. High discrimination alone is therefore insufficient to justify routine clinical implementation. While waiting for large-scale prospective validation, practicing clinicians should adopt a pragmatic strategy: utilizing standardized NCCT scoring systems (combining clinical markers such as GCS and SBP with validated imaging signs) as the baseline, while strictly adhering to the multidisciplinary protocols outlined in the CODE-ICH consensus to minimize time-to-treatment. Future research must shift focus from merely improving AUC values to designing lightweight, interpretable models that can be seamlessly deployed in the emergency setting to close the gap between prediction and patient benefit. These requirements can be organized as a staged translational pathway from model development and internal validation to external validation, prospective clinical evaluation, and eventual clinical integration (Table 6). Each stage addresses a distinct source of uncertainty, and progression toward routine implementation requires increasingly strong evidence of model robustness, transportability, clinical utility, and workflow feasibility.

**Table 6:** Translational pathway for HE prediction models from model development to clinical integration.

Stage	Primary objective	Key evaluation requirements	Major challenges in HE prediction
Model development	Develop a reproducible model for individualized HE risk estimation	Clear target population and HE definition; appropriate feature selection; handling of missing data; control of overfitting; transparent reporting	Small datasets; heterogeneous HE definitions; variable imaging acquisition; manual segmentation and feature instability
Internal validation	Estimate model optimism and stability within the development setting	Bootstrap or cross-validation; discrimination; calibration; uncertainty estimates	Optimistic performance in small or highly selected cohorts; inadequate assessment beyond AUC
External validation	Assess transportability to independent populations	Geographic or temporal external cohorts; discrimination and calibration; assessment across scanners, institutions, and patient case mix	Domain shift; scanner/protocol variability; differences in HE prevalence and case mix
Prospective clinical evaluation	Determine real-world clinical utility and workflow feasibility	Prespecified decision thresholds; net benefit/DCA; turnaround time; failure-mode analysis; prospective workflow evaluation	Hyperacute time constraints; IT integration; model interpretability; uncertain actionable thresholds
Clinical integration	Determine whether model-guided care improves clinical decision-making and outcomes	Prospective impact studies; treatment-selection effects; workflow efficiency; clinician acceptance; patient-centered outcomes and safety	Lack of proven treatment-impact thresholds; integration into multidisciplinary care; accountability, generalizability, and long-term monitoring

HE, hematoma expansion; AUC, area under the receiver operating characteristic curve; DCA, decision-curve analysis.

In parallel with this clinical translation pathway, the technical architecture of HE prediction models has also evolved rapidly, as summarized in Figure 3. The evolution of predictive frameworks for HE reflects a paradigm shift from simple linear associations to complex generative modeling. The foundational era relied on base models (e.g., LR and SVM) and feature engineering. While these methods established early benchmarks, their performance depended strongly on prespecified or handcrafted feature representations, model specification, and the size and quality of the available datasets. The advent of DL, particularly CNNs and transformers, marked a transition to “feature learning,” enabling the automated extraction of high-dimensional representations directly from raw CT data.

**Figure 3:**
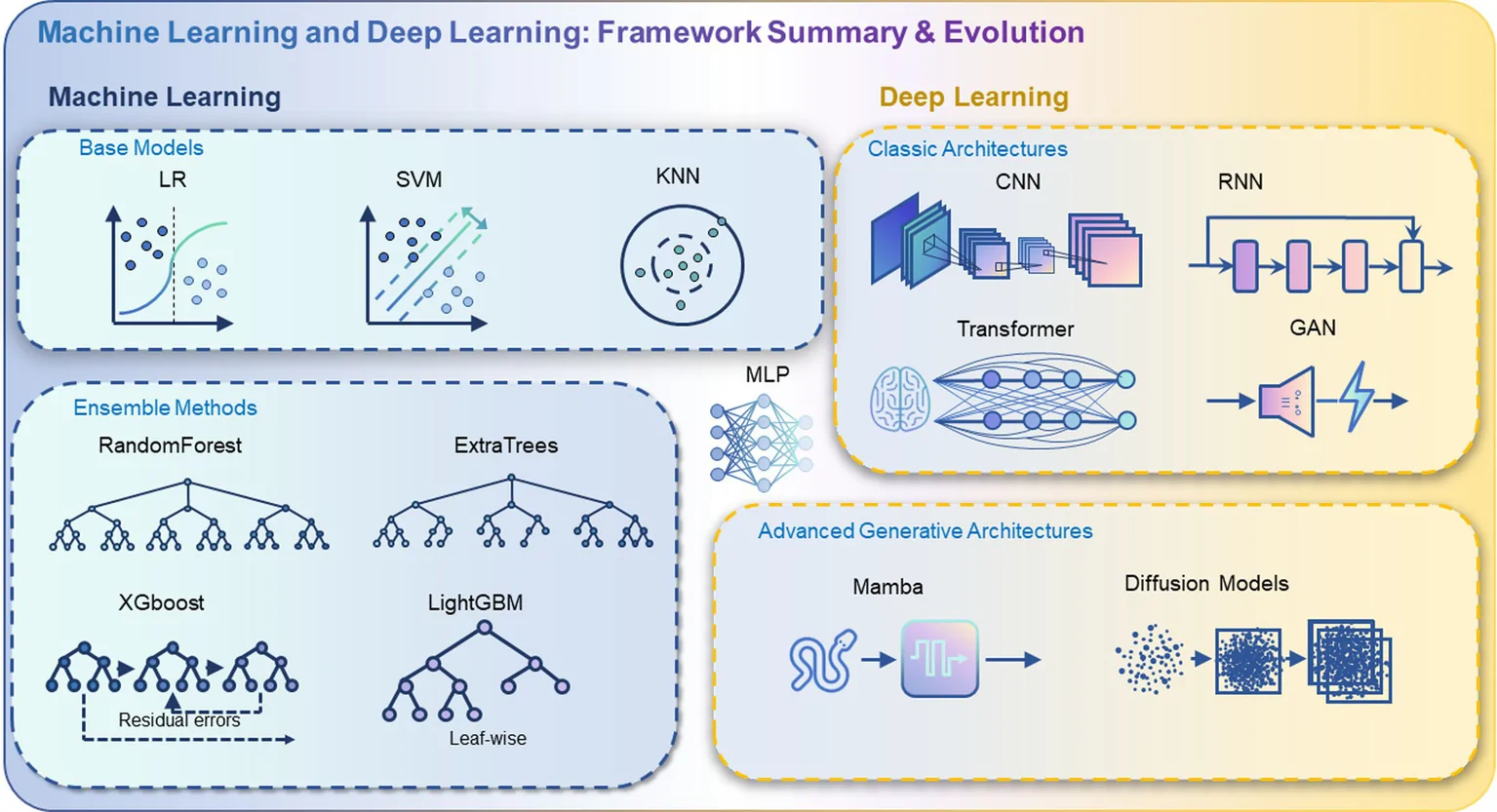
Evolution of predictive frameworks for HE. Conceptual overview illustrating the progression of predictive strategies for HE in ICH. Early approaches relied on individual clinical or imaging predictors and conventional statistical models. Subsequent developments incorporated clinical scoring systems and radiomics-based machine learning models that extract quantitative imaging features. More recent frameworks utilize deep learning architectures and multimodal data integration to enable automated feature learning and improved predictive performance. Emerging generative models may further enhance model robustness by addressing data scarcity and heterogeneity.

However, even these advanced approaches are constrained by small sample sizes, limited multicenter validation, heterogeneity in imaging acquisition, ROI delineation, and the subjective nature of manual segmentation (Gregório et al., 2018). The reliance on large, annotated datasets remains a critical bottleneck as medical imaging data are inherently scarce and expensive to label.

Emerging architectures, including state-space models and diffusion-based generative approaches, are increasingly being explored in medical imaging and may offer future opportunities for HE modeling, although HE-specific evidence remains limited (Figure 3, right panel). To solve the efficiency trade-off found in transformers, Mamba architectures utilize selective state space models (SSMs). With linear computational complexity (*O(N)*), Mamba allows for the modeling of global, long-range dependencies across the entire brain volume without the memory bottlenecks of previous architectures (Gu and Dao, 2024; Ma et al., 2024). This efficiency is crucial for developing fully automated pipelines that eliminate the subjectivity of manual ROI delineation. To tackle the constraints of small sample sizes, diffusion models provide a robust mechanism for data augmentation. By generating high-fidelity, synthetic 3D CT images, these models can enrich training datasets and simulate rare heterogeneous morphologies, thereby improving algorithm robustness against domain shifts (Pinaya et al., 2022). Ultimately, technical innovation must be coupled with rigorous validation. Future research should prioritize large-scale, standardized, multicenter studies, integration of multimodal data, and algorithm optimization to facilitate clinical translation. With these advancements, precise and timely HE prediction has the potential to become a practical clinical tool, which could guide personalized treatment and improve outcomes in patients with ICH.

Secondary analyses of major ICH trials have also provided opportunities to examine whether clinical or imaging biomarkers identify subgroups with differential responses to anti-expansion therapies. To date, however, evidence that conventional NCCT markers reliably identify treatment-responsive subgroups for TXA or intensive blood pressure lowering remains inconsistent. These observations reinforce the need to evaluate prediction models not only for their association with subsequent HE but also for their ability to support reproducible treatment selection in prospectively designed studies.

Several limitations of this review should also be acknowledged. Because this is a narrative review, literature selection was not conducted according to a prespecified systematic review protocol, and no formal study-level risk-of-bias assessment or quantitative meta-analysis was performed. Therefore, incomplete literature capture and selective citation bias cannot be excluded.

Overall, HE is a common, early, and complex complication of ICH, and accurate HE prediction is critical for achieving precise intervention and improving patient prognosis. Multiple predictors of HE have been identified, and the integration of clinical variables, imaging biomarkers, radiomics, and AI-based methods has expanded the range of quantitative approaches available for HE risk stratification. Future research should prioritize independent external validation, prospective evaluation, calibration, interpretability, and formal assessment of clinical utility before these models are incorporated into routine treatment pathways.
